# (*E*)-3-(3,5-Dimeth­oxy­phen­yl)-1-(1-hy­droxy­naphthalen-2-yl)prop-2-en-1-one

**DOI:** 10.1107/S1600536812046715

**Published:** 2012-11-24

**Authors:** Ha-Jin Lee, Yoongho Lim, Dongsoo Koh

**Affiliations:** a Jeonju Center, Korea Basic Science Center (KBSI), Jeonju 561-765, Republic of Korea; b Division of Bioscience and Biotechnology, BMIC, Konkuk University, Seoul 143-701, Republic of Korea; c Department of Applied Chemistry, Dongduk Women’s University, Seoul 136-714, Republic of Korea

## Abstract

In the title mol­ecule, C_21_H_18_O_4_, the C=C bond of the central enone group adopts a *trans* conformation. The dihedral angle formed by the naphthalene ring system and the benzene ring is 2.97 (11)°. The hy­droxy group is involved in an intra­molecular O—H⋯O hydrogen bond. In the crystal, weak C—H⋯O hydrogen bonds link the mol­ecules into chains along [001].

## Related literature

For the synthesis and biological properties of chalcone derivatives, see: Sharma *et al.* (2012 ▶); Singh *et al.* (2012 ▶); Bandgar *et al.* (2010 ▶); Hans *et al.* (2010 ▶); Hwang *et al.* (2011 ▶). For related structures, see: Fadzillah *et al.* (2012 ▶); Jasinski *et al.* (2011 ▶). For standard bond lengths, see: Allen *et al.* (1987 ▶).
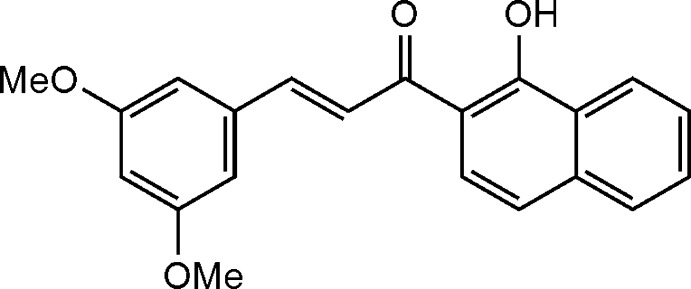


## Experimental

### 

#### Crystal data

C_21_H_18_O_4_
*M* *_r_* = 334.35
Orthorhombic, 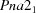

*a* = 30.179 (3) Å
*b* = 3.9127 (3) Å
*c* = 13.7363 (12) Å
*V* = 1622.0 (2) Å^3^
*Z* = 4
Mo *K*α radiation
μ = 0.09 mm^−1^
*T* = 200 K
0.24 × 0.22 × 0.17 mm

#### Data collection

Bruker SMART CCD diffractometer
11095 measured reflections
3479 independent reflections
1828 reflections with *I* > 2σ(*I*)
*R*_int_ = 0.064

#### Refinement

*R*[*F* ^2^ > 2σ(*F* ^2^)] = 0.044
*wR*(*F* ^2^) = 0.102
*S* = 0.94
3479 reflections
229 parameters
1 restraint
H-atom parameters constrained
Δρ_max_ = 0.22 e Å^−3^
Δρ_min_ = −0.23 e Å^−3^

### 

Data collection: *SMART* (Bruker, 2000 ▶); cell refinement: *SAINT* (Bruker, 2000 ▶); data reduction: *SAINT*; program(s) used to solve structure: *SHELXS97* (Sheldrick, 2008 ▶); program(s) used to refine structure: *SHELXL97* (Sheldrick, 2008 ▶); molecular graphics: *PLATON* (Spek, 2009 ▶); software used to prepare material for publication: *SHELXTL* (Sheldrick, 2008 ▶).

## Supplementary Material

Crystal structure: contains datablock(s) I, global. DOI: 10.1107/S1600536812046715/lh5555sup1.cif

Structure factors: contains datablock(s) I. DOI: 10.1107/S1600536812046715/lh5555Isup2.hkl

Supplementary material file. DOI: 10.1107/S1600536812046715/lh5555Isup3.cml

Additional supplementary materials:  crystallographic information; 3D view; checkCIF report

## Figures and Tables

**Table 1:** Hydrogen-bond geometry (Å, °)

*D*—H⋯*A*	*D*—H	H⋯*A*	*D*⋯*A*	*D*—H⋯*A*
O4—H4⋯O1	0.84	1.75	2.503 (3)	147
C7—H7*C*⋯O1^i^	0.98	2.59	3.157 (4)	117
C10—H10*B*⋯O2^i^	0.98	2.54	3.344 (4)	139

Symmetry code: (i) 
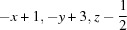
.

**Fig. 1.**
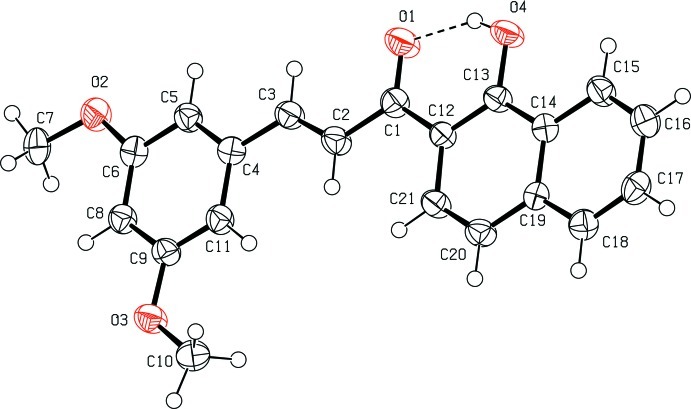
The molecular structure of the title compound, showing displacement ellipsoids drawn at the 50% probability level. The dashed line indicates a hydrogen bond.

**Fig. 2.**
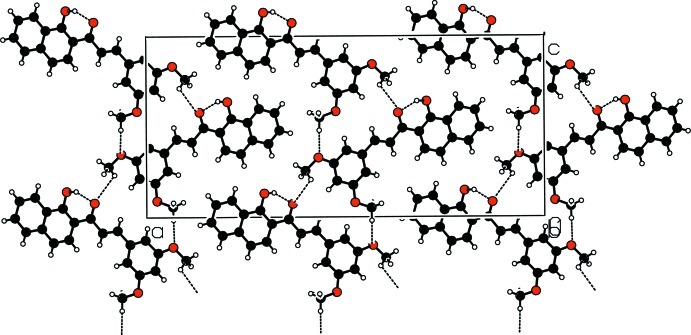
Part of the crystal structure with weak intermolecular C—H···O hydrogen bonds shown as dashed lines.

## supplementary crystallographic information

### Comment

Chalcones are one of the secondary metabolites in plants and belong to a flavonoid class with a C6—C3—C6 skeleton and C3 skeleton which is an α,β-unsaturated carbonyl (enone) group. Because of their diverse biological activities including anticancer (Singh *et al.*, 2012), anti-inflammatory (Bandgar *et al.*, 2010), anti-tubercular (Hans *et al.*, 2010), and antimicrobial (Sharma *et al.*, 2012), various chlacones have been isolated from natural sources and syntheized. In continuation of our research to develop novel chalcone derivatives which show broad range of biological activities (Hwang *et al.*, 2011) the title compound was synthesized and and its crystal structure was determined.

The molecular structure of the title compound is shown in Fig .1. The C2═C3 bond of the central enone group adopts a *trans* configuration. The dihedral angle formed by the naphthalene ring system and the benzene ring is 2.97 (11)°. The C1═O1 bond [1.242 (3) Å] is slightly longer than the standard value (Allen *et al.* 1987) as this group is involved in an intramolecular O—H···O hydrogen bond with the hydroxy group. In the crystal, weak C—H···O hydrogen bonds link the molecules into one-dimensional chains along [001] (Fig. 2). Examples of structures of substituted prop-2-en-1-one compounds have been published (Fadzillah *et al.*, 2012; Jasinski *et al.*, 2011).

### Experimental

A solution of 1-hydroxy-2-acetonaphthone (186 mg, 1 mmol) and 3,5-dimethoxybenzaldehyde (166 mg, 1 mmol) was dissolved in 10 ml of ethanol and the temperature was adjusted to around 276-277K in an ice-bath. To the cooled reaction mixture was added 0.5 ml of 50% aqueous KOH solution, and the reaction mixture was stirred at room temperature for 24 h. This mixture was poured into iced water (20 ml) was acidified with 6 N HCl solution. The mixture was extracted with ethylacetate (3 × 20 ml) and the combined organic layers were dried under MgSO_4_. Filtration and evaporation of the filtrate gave a residue which was purified by flash chromatography to give the title compound (210 mg, 63%). Recrystallization of the title compound in ethanol gave orange colored crystals (mp: 422-424K).

### Refinement

H atoms were placed in calculated positions and refined as riding with C—H = 0.95-0.98Å, O—H = 0.84 Å and *U*_iso_(H) = 1.2 *U*_eq_(C) or *U*_iso_(H) = 1.5*U*_eq_(C_methyl_, O).

### Crystal data

**Table:**

C_21_H_18_O_4_	*F*(000) = 704
*M**_r_* = 334.35	*D*_x_ = 1.369 Mg m^−^^3^
Orthorhombic, *P**n**a*2_1_	Mo *K*α radiation, λ = 0.71073 Å
Hall symbol: P 2c -2n	Cell parameters from 2578 reflections
*a* = 30.179 (3) Å	θ = 2.7–27.8°
*b* = 3.9127 (3) Å	µ = 0.09 mm^−^^1^
*c* = 13.7363 (12) Å	*T* = 200 K
*V* = 1622.0 (2) Å^3^	Block, orange
*Z* = 4	0.24 × 0.22 × 0.17 mm

### Data collection

**Table:**

Bruker SMART CCD diffractometer	1828 reflections with *I* > 2σ(*I*)
Radiation source: fine-focus sealed tube	*R*_int_ = 0.064
Graphite monochromator	θ_max_ = 28.3°, θ_min_ = 1.4°
φ and ω scans	*h* = −32→40
11095 measured reflections	*k* = −5→5
3479 independent reflections	*l* = −16→18

### Refinement

**Table:**

Refinement on *F*^2^	Primary atom site location: structure-invariant direct methods
Least-squares matrix: full	Secondary atom site location: difference Fourier map
*R*[*F*^2^ > 2σ(*F*^2^)] = 0.044	Hydrogen site location: inferred from neighbouring sites
*wR*(*F*^2^) = 0.102	H-atom parameters constrained
*S* = 0.94	*w* = 1/[σ^2^(*F*_o_^2^) + (0.0276*P*)^2^] where *P* = (*F*_o_^2^ + 2*F*_c_^2^)/3
3479 reflections	(Δ/σ)_max_ < 0.001
229 parameters	Δρ_max_ = 0.22 e Å^−^^3^
1 restraint	Δρ_min_ = −0.23 e Å^−^^3^

### Special details

**Table:**

Geometry. All e.s.d.'s (except the e.s.d. in the dihedral angle between two l.s. planes) are estimated using the full covariance matrix. The cell e.s.d.'s are taken into account individually in the estimation of e.s.d.'s in distances, angles and torsion angles; correlations between e.s.d.'s in cell parameters are only used when they are defined by crystal symmetry. An approximate (isotropic) treatment of cell e.s.d.'s is used for estimating e.s.d.'s involving l.s. planes.
Refinement. Refinement of *F*^2^ against ALL reflections. The weighted *R*-factor *wR* and goodness of fit *S* are based on *F*^2^, conventional *R*-factors *R* are based on *F*, with *F* set to zero for negative *F*^2^. The threshold expression of *F*^2^ > σ(*F*^2^) is used only for calculating *R*-factors(gt) *etc*. and is not relevant to the choice of reflections for refinement. *R*-factors based on *F*^2^ are statistically about twice as large as those based on *F*, and *R*- factors based on ALL data will be even larger.

### Fractional atomic coordinates and isotropic or equivalent isotropic displacement parameters (Å^2^)

**Table:**

	*x*	*y*	*z*	*U*_iso_*/*U*_eq_	
C1	0.35700 (10)	0.8619 (7)	0.4892 (2)	0.0348 (7)	
O1	0.36736 (7)	0.9296 (6)	0.57454 (14)	0.0485 (6)	
C2	0.38738 (10)	0.9586 (8)	0.4096 (2)	0.0362 (8)	
H2	0.3791	0.9009	0.3449	0.043*	
C3	0.42532 (10)	1.1203 (7)	0.4228 (2)	0.0352 (7)	
H3	0.4326	1.1809	0.4878	0.042*	
C4	0.45715 (10)	1.2153 (7)	0.3474 (2)	0.0312 (7)	
C5	0.49588 (10)	1.3745 (7)	0.3726 (2)	0.0340 (7)	
H5	0.5011	1.4326	0.4388	0.041*	
C6	0.52799 (10)	1.4530 (7)	0.3019 (2)	0.0335 (8)	
O2	0.56629 (7)	1.5970 (6)	0.33591 (14)	0.0424 (6)	
C7	0.59853 (11)	1.7037 (8)	0.2650 (2)	0.0420 (9)	
H7A	0.6101	1.5029	0.2307	0.063*	
H7B	0.6229	1.8220	0.2979	0.063*	
H7C	0.5846	1.8587	0.2182	0.063*	
C8	0.52006 (10)	1.3789 (7)	0.2050 (2)	0.0334 (7)	
H8	0.5414	1.4332	0.1565	0.040*	
C9	0.48010 (10)	1.2229 (7)	0.1799 (2)	0.0322 (7)	
O3	0.47555 (7)	1.1594 (5)	0.08193 (15)	0.0416 (5)	
C10	0.43530 (10)	1.0023 (8)	0.0509 (2)	0.0428 (8)	
H10A	0.4337	0.7696	0.0771	0.064*	
H10B	0.4345	0.9936	−0.0204	0.064*	
H10C	0.4100	1.1357	0.0746	0.064*	
C11	0.44877 (10)	1.1405 (7)	0.2483 (2)	0.0343 (8)	
H11	0.4218	1.0346	0.2293	0.041*	
C12	0.31508 (10)	0.6927 (7)	0.4670 (2)	0.0290 (7)	
C13	0.28636 (10)	0.6041 (8)	0.5424 (2)	0.0319 (7)	
O4	0.29656 (7)	0.6743 (6)	0.63545 (14)	0.0437 (6)	
H4	0.3204	0.7843	0.6375	0.066*	
C14	0.24525 (10)	0.4418 (7)	0.5241 (2)	0.0311 (7)	
C15	0.21607 (10)	0.3500 (8)	0.6006 (2)	0.0377 (8)	
H15	0.2241	0.3941	0.6663	0.045*	
C16	0.17643 (10)	0.1979 (8)	0.5797 (2)	0.0408 (8)	
H16	0.1568	0.1392	0.6311	0.049*	
C17	0.16454 (10)	0.1281 (8)	0.4837 (2)	0.0398 (8)	
H17	0.1368	0.0242	0.4702	0.048*	
C18	0.19236 (11)	0.2075 (8)	0.4093 (2)	0.0366 (8)	
H18	0.1841	0.1533	0.3444	0.044*	
C19	0.23315 (10)	0.3683 (7)	0.4268 (2)	0.0293 (7)	
C20	0.26243 (10)	0.4564 (7)	0.3506 (2)	0.0360 (8)	
H20	0.2545	0.4061	0.2852	0.043*	
C21	0.30156 (10)	0.6117 (8)	0.3696 (2)	0.0351 (8)	
H21	0.3206	0.6686	0.3170	0.042*	

### Atomic displacement parameters (Å^2^)

**Table:**

	*U*^11^	*U*^22^	*U*^33^	*U*^12^	*U*^13^	*U*^23^
C1	0.0311 (19)	0.0388 (19)	0.0344 (19)	−0.0019 (15)	−0.0011 (14)	0.0023 (16)
O1	0.0434 (15)	0.0720 (17)	0.0302 (13)	−0.0130 (12)	−0.0004 (10)	−0.0007 (12)
C2	0.0288 (19)	0.049 (2)	0.0312 (18)	−0.0061 (15)	0.0035 (13)	−0.0010 (15)
C3	0.035 (2)	0.0414 (19)	0.0297 (16)	−0.0024 (16)	−0.0001 (15)	0.0006 (16)
C4	0.0289 (18)	0.0324 (18)	0.0324 (18)	0.0014 (14)	−0.0023 (14)	0.0011 (14)
C5	0.0316 (19)	0.0379 (19)	0.0324 (17)	−0.0025 (15)	0.0020 (14)	0.0014 (15)
C6	0.0286 (19)	0.0322 (18)	0.0396 (19)	−0.0001 (14)	−0.0033 (14)	0.0036 (15)
O2	0.0314 (13)	0.0531 (14)	0.0428 (14)	−0.0114 (11)	−0.0010 (11)	0.0022 (11)
C7	0.031 (2)	0.043 (2)	0.052 (2)	−0.0077 (16)	0.0043 (16)	0.0082 (17)
C8	0.0286 (19)	0.0368 (18)	0.0348 (18)	−0.0012 (14)	0.0022 (14)	0.0004 (15)
C9	0.0363 (19)	0.0325 (17)	0.0279 (18)	0.0032 (14)	−0.0055 (14)	0.0035 (14)
O3	0.0376 (14)	0.0555 (15)	0.0316 (12)	−0.0060 (11)	−0.0007 (10)	−0.0017 (11)
C10	0.042 (2)	0.049 (2)	0.0380 (19)	−0.0040 (17)	−0.0094 (15)	−0.0001 (15)
C11	0.0290 (19)	0.040 (2)	0.0342 (18)	−0.0053 (15)	0.0003 (14)	0.0021 (15)
C12	0.0258 (18)	0.0345 (18)	0.0266 (16)	−0.0016 (14)	−0.0005 (13)	0.0018 (14)
C13	0.0304 (19)	0.0373 (18)	0.0281 (17)	−0.0001 (14)	0.0012 (14)	−0.0002 (15)
O4	0.0414 (16)	0.0659 (16)	0.0237 (12)	−0.0129 (12)	−0.0014 (10)	−0.0015 (11)
C14	0.0303 (19)	0.0304 (16)	0.0326 (18)	0.0000 (14)	0.0012 (13)	0.0024 (14)
C15	0.036 (2)	0.045 (2)	0.0329 (19)	−0.0040 (15)	0.0036 (14)	0.0042 (15)
C16	0.035 (2)	0.045 (2)	0.042 (2)	−0.0020 (16)	0.0060 (16)	0.0032 (17)
C17	0.029 (2)	0.0414 (19)	0.049 (2)	−0.0073 (15)	0.0001 (16)	−0.0013 (17)
C18	0.034 (2)	0.038 (2)	0.0383 (19)	−0.0013 (15)	−0.0026 (14)	0.0020 (15)
C19	0.0282 (18)	0.0269 (16)	0.0329 (16)	−0.0012 (13)	0.0003 (14)	0.0009 (14)
C20	0.035 (2)	0.0434 (19)	0.0293 (17)	−0.0006 (15)	−0.0047 (14)	−0.0002 (15)
C21	0.0327 (19)	0.045 (2)	0.0279 (17)	−0.0018 (15)	0.0016 (14)	0.0014 (15)

### Geometric parameters (Å, º)

**Table:**

C1—O1	1.242 (3)	C10—H10B	0.9800
C1—C12	1.460 (4)	C10—H10C	0.9800
C1—C2	1.476 (4)	C11—H11	0.9500
C2—C3	1.321 (4)	C12—C13	1.395 (4)
C2—H2	0.9500	C12—C21	1.433 (4)
C3—C4	1.460 (4)	C13—O4	1.343 (3)
C3—H3	0.9500	C13—C14	1.416 (4)
C4—C5	1.369 (4)	O4—H4	0.8400
C4—C11	1.416 (4)	C14—C19	1.414 (4)
C5—C6	1.406 (4)	C14—C15	1.418 (4)
C5—H5	0.9500	C15—C16	1.367 (4)
C6—O2	1.368 (3)	C15—H15	0.9500
C6—C8	1.384 (4)	C16—C17	1.394 (4)
O2—C7	1.438 (3)	C16—H16	0.9500
C7—H7A	0.9800	C17—C18	1.359 (4)
C7—H7B	0.9800	C17—H17	0.9500
C7—H7C	0.9800	C18—C19	1.403 (4)
C8—C9	1.395 (4)	C18—H18	0.9500
C8—H8	0.9500	C19—C20	1.413 (4)
C9—C11	1.371 (4)	C20—C21	1.354 (4)
C9—O3	1.375 (3)	C20—H20	0.9500
O3—C10	1.427 (3)	C21—H21	0.9500
C10—H10A	0.9800		
			
O1—C1—C12	120.8 (3)	H10A—C10—H10C	109.5
O1—C1—C2	119.2 (3)	H10B—C10—H10C	109.5
C12—C1—C2	120.0 (3)	C9—C11—C4	119.2 (3)
C3—C2—C1	124.1 (3)	C9—C11—H11	120.4
C3—C2—H2	118.0	C4—C11—H11	120.4
C1—C2—H2	118.0	C13—C12—C21	117.5 (3)
C2—C3—C4	126.5 (3)	C13—C12—C1	119.7 (3)
C2—C3—H3	116.7	C21—C12—C1	122.8 (3)
C4—C3—H3	116.7	O4—C13—C12	120.9 (3)
C5—C4—C11	119.3 (3)	O4—C13—C14	117.5 (2)
C5—C4—C3	119.9 (3)	C12—C13—C14	121.6 (3)
C11—C4—C3	120.8 (3)	C13—O4—H4	109.5
C4—C5—C6	120.9 (3)	C19—C14—C13	119.0 (3)
C4—C5—H5	119.6	C19—C14—C15	119.2 (3)
C6—C5—H5	119.6	C13—C14—C15	121.7 (3)
O2—C6—C8	124.1 (3)	C16—C15—C14	119.9 (3)
O2—C6—C5	115.9 (3)	C16—C15—H15	120.0
C8—C6—C5	120.0 (3)	C14—C15—H15	120.0
C6—O2—C7	117.4 (2)	C15—C16—C17	120.6 (3)
O2—C7—H7A	109.5	C15—C16—H16	119.7
O2—C7—H7B	109.5	C17—C16—H16	119.7
H7A—C7—H7B	109.5	C18—C17—C16	120.5 (3)
O2—C7—H7C	109.5	C18—C17—H17	119.7
H7A—C7—H7C	109.5	C16—C17—H17	119.7
H7B—C7—H7C	109.5	C17—C18—C19	121.0 (3)
C6—C8—C9	118.6 (3)	C17—C18—H18	119.5
C6—C8—H8	120.7	C19—C18—H18	119.5
C9—C8—H8	120.7	C18—C19—C20	122.0 (3)
C11—C9—O3	124.0 (3)	C18—C19—C14	118.7 (3)
C11—C9—C8	122.0 (3)	C20—C19—C14	119.3 (3)
O3—C9—C8	114.0 (3)	C21—C20—C19	120.8 (3)
C9—O3—C10	117.1 (2)	C21—C20—H20	119.6
O3—C10—H10A	109.5	C19—C20—H20	119.6
O3—C10—H10B	109.5	C20—C21—C12	121.9 (3)
H10A—C10—H10B	109.5	C20—C21—H21	119.1
O3—C10—H10C	109.5	C12—C21—H21	119.1
			
O1—C1—C2—C3	−1.1 (5)	C21—C12—C13—O4	−179.4 (3)
C12—C1—C2—C3	178.1 (3)	C1—C12—C13—O4	0.5 (4)
C1—C2—C3—C4	178.4 (3)	C21—C12—C13—C14	−0.3 (4)
C2—C3—C4—C5	−178.1 (3)	C1—C12—C13—C14	179.6 (3)
C2—C3—C4—C11	1.0 (5)	O4—C13—C14—C19	179.1 (3)
C11—C4—C5—C6	−2.3 (5)	C12—C13—C14—C19	0.0 (4)
C3—C4—C5—C6	176.8 (3)	O4—C13—C14—C15	−1.2 (4)
C4—C5—C6—O2	−177.0 (3)	C12—C13—C14—C15	179.7 (3)
C4—C5—C6—C8	2.0 (4)	C19—C14—C15—C16	−1.2 (5)
C8—C6—O2—C7	5.5 (4)	C13—C14—C15—C16	179.1 (3)
C5—C6—O2—C7	−175.6 (3)	C14—C15—C16—C17	0.8 (5)
O2—C6—C8—C9	178.2 (3)	C15—C16—C17—C18	0.6 (5)
C5—C6—C8—C9	−0.7 (4)	C16—C17—C18—C19	−1.5 (5)
C6—C8—C9—C11	−0.3 (4)	C17—C18—C19—C20	−179.3 (3)
C6—C8—C9—O3	−179.8 (3)	C17—C18—C19—C14	1.0 (4)
C11—C9—O3—C10	0.9 (4)	C13—C14—C19—C18	180.0 (3)
C8—C9—O3—C10	−179.6 (2)	C15—C14—C19—C18	0.3 (4)
O3—C9—C11—C4	179.4 (3)	C13—C14—C19—C20	0.4 (4)
C8—C9—C11—C4	−0.1 (4)	C15—C14—C19—C20	−179.3 (3)
C5—C4—C11—C9	1.3 (5)	C18—C19—C20—C21	179.9 (3)
C3—C4—C11—C9	−177.8 (3)	C14—C19—C20—C21	−0.4 (4)
O1—C1—C12—C13	−0.4 (4)	C19—C20—C21—C12	0.1 (4)
C2—C1—C12—C13	−179.6 (3)	C13—C12—C21—C20	0.3 (4)
O1—C1—C12—C21	179.5 (3)	C1—C12—C21—C20	−179.6 (3)
C2—C1—C12—C21	0.3 (4)		

### Hydrogen-bond geometry (Å, º)

**Table:**

*D*—H···*A*	*D*—H	H···*A*	*D*···*A*	*D*—H···*A*
O4—H4···O1	0.84	1.75	2.503 (3)	147
C7—H7*C*···O1^i^	0.98	2.59	3.157 (4)	117
C10—H10*B*···O2^i^	0.98	2.54	3.344 (4)	139

Symmetry code: (i) −*x*+1, −*y*+3, *z*−1/2.
